# Serum proteomics identify potential biomarkers for nasopharyngeal carcinoma sensitivity to radiotherapy

**DOI:** 10.1042/BSR20190027

**Published:** 2019-05-14

**Authors:** Guangying Zhang, Kun Zhang, Chao Li, Yanyan Li, Zhanzhan Li, Na Li, Qin Zhou, Liangfang Shen

**Affiliations:** 1 Department of Oncology, Xiangya Hospital, Central South University, Changsha, Hunan Province 410008, China; 2 Department of Outpatient, Xiangya Hospital, Central South University, Changsha, Hunan Province 410008, China

**Keywords:** biomarkers, Nasopharyngeal carcinoma, radioresistance, serum proteomics

## Abstract

Radiotherapy is the primary treatment option for nasopharyngeal carcinoma (NPC). Local recurrence and metastasis caused by radioresistance become a bottleneck of curative effect for patients with NPC. Currently, serum predictive biomarkers of radioresistance are scare. We enrolled NPC patients, who underwent radiotherapy in the Department of Oncology, Xiangya Hospital, Central Southern University, and analyzed the serum proteins profiles in NPC patients using with quantitative label-free proteomics using ultra-definition MS. Patients were divided into those who were radioresistant and radiosensitive by the overall reduction (≤50% or >50%, respectively) in tumor extent. The MS/MS spectrum database search identified 911 proteins and 809 proteins are quantitatable. Eight proteins significantly up-regulated and 12 serum proteins were significantly down-regulated in the radioresistance group compared with radiosensitivity group (*P*<0.05). Finally, five proteins entered the optimal models, including secreted protein acidic and cysteine rich (SPARC) (*P* *=*0.032), serpin family D member 1S (ERPIND1) (*P* *=*0.040), complement C4B (C4B) (*P* *=*0.017), peptidylprolyl Isomerase B (PPIB) (*P* *=*0.042), and family with sequence similarity 173 member A (FAM173A) (*P* *=*0.017). In all patient, the area under the curves (AUC) for SPARC, SERPIND, C4B, PPIB, and FAM173A were 0.716 (95% CI: 0.574–0.881), 0.697 (95% CI: 0.837–0.858), 0.686 (95% CI: 0.522–0.850), 0.668 (95% CI: 0.502–0.834) and 0.657 (95% CI: 0.512–0.825), respectively. The AUC of five selected proteins was 0.968 (95% CI: 0.918–1.000) with the sensitivity of 0.941 and the specificity of 0.926. Our result indicated that a panel including five serum protein (SPARC SERPIND1 C4B PPIB FAM173A) based on serum proteomics provided a high discrimination ability for radiotherapy effects in NPC patients. Studies with larger sample size and longer follow-up outcome are required.

## Introduction

Nasopharyngeal carcinoma (NPC) is an Epstein–Barr (EB) virus-associated epithelial malignancy exhibiting unusual ethnic and geographical distributions [1,2]. NPC is a typical regional disease; most patients live in Southern Asia (particularly Southeast China) [3]. The incidence ranges from 30 to 50 cases per 10,000 of the population [4]. NPC is highly malignant; both lymph node and distant metastases may develop early, without obvious symptoms. Most patients have middle- and late-stage disease when diagnosed [5]. Radiation therapy is the major therapeutic modality used to treat NPC, and most NPC patients can be cured if the disease is diagnosed and treated at an early stage. The 5-year survival rate of patients with stages I–IVB NPC is approximately 85% [6]. However, some patients do not benefit from radiotherapy due to radioresistance caused by local recurrence and distant metastasis [7]. Radio resistance and radiation-induced cell damage are affected by the treatment methods employed and genetic differences in cell cycle regulation, apoptotic/anti-apoptotic mechanisms, and DNA repair pathways [8,9]. However, the details remain unclear. A previous study used a microarray to identify certain NPC radioresistance genes, but the data were not confirmed in other studies [10–12]. It is likely that the data reflect tissue specificities rather than radioresistance mechanisms. New techniques enable the exploration of tumor genetic profiles. To date, the molecular mechanism of radioresistance remains unclear; NPC is caused by many different factors, and few serum biomarkers predictive of radioresistance are known [13]. Besides, the present treatment guidelines have certain limitations, and treatments do not vary by tumor stage or lymph node metastasis status. However, histological staging and molecular subtyping have shown that treatment sensitivity is affected by stage [14,15]. Thus, treatment should be patient specific, and biomarkers of radiotherapeutic sensitivity are urgently required. Finding biomarkers of radiation sensitivity would not only benefit the individual but also reduce healthcare costs. Previous study showed that occurrence and development process of NPC involved many tumor-associated proteins [16]. Protein overexpression led by related gene abnormality in serum cannot only reflect the degree of malignancy but also lead to dynamic changes of protein expression after the excision of primary lesion and radiotherapy, indicating that abnormal expression of certain proteins was associated with NPC treatment and prognosis [17]. Surface-enhanced laser desorption/ionization time-of-flight mass spectrometry renders serum proteomics possible. Here, we analyzed the serum proteins of NPC patients who were either radiosensitive or radioresistant to build a diagnostic panel of prognosis after radiotherapy.

## Materials and methods

### Patients and blood samples

We enrolled NPC patients who underwent radiotherapy in the Department of Oncology, Xiangya Hospital, Central Southern University, China, from April to June 2018. The selection criteria were pathologically confirmed NPC; no prior radiotherapy, chemotherapy, surgery, adjuvant therapy, or anticarcinoma drug therapy; and stage III or IV disease. Serum samples were collected prior to treatment; all patients were over 18 years of age. The exclusion criteria were any severe infection; any other tumor; any severe liver, kidney, immune system, or hematological disease; and/or any prior cancer treatment. Blood samples were collected into tubes containing EDTA and an antithrombotic agent and stored at –80°C. The study was approved by the ethics committee of Xiangya Hospital, Central Southern University. The research has been carried out in accordance with the World Medical Association Declaration of Helsinki, and that all subjects provided written informed consent.

### Clinical data

We extracted clinical data from electronic medical records. We recorded gender, age, cancer type, American Joint Committee on Cancer stage, nasopharyngeal gross tumor volume (GTV), lymph node GTV prior to treatment, lymph node remission rate, overall tumor reduction rate, routine blood data (the numbers of red and white blood cells, neutrophils, and lymphocytes), and the levels of hemoglobin, α-fetoprotein, carcinogenic embryonic antigen, CA125, CA242, C19F, EB virus DNA, EAIgA, and EBVCA IgA. Tumor biomarkers were quantitated using a chemiluminescence method (Immulite; DPC, U.S.A.). The EB virus DNA was quantitated via fluorescence-PCR. Treatment included induction chemotherapy (docetaxel 120–164 m^−2^ and cisplatin 60–120 m^−2^ on days 1–2) and concurrent chemotherapy (cisplatin 65–120 m^−2^ on days 1–2) and radiotherapy (70.4 Gy/2.2 Gy over 32 fractions [PGTVnx], 70.4 Gy/2.2Gy over 32 fractions [PGTVnd], 60.8 Gy/1.9 Gy over 32 fractions [PTV1], and 54 Gy/1.8 Gy over 30 fractions [PTV2]). Radiotherapy sensitivity was then evaluated as the reduction in the maximum cross-sectional area of the primary lesion, calculated as the maximum transverse diameter × the maximum vertical length.

### Experimental

**Figure 1:**
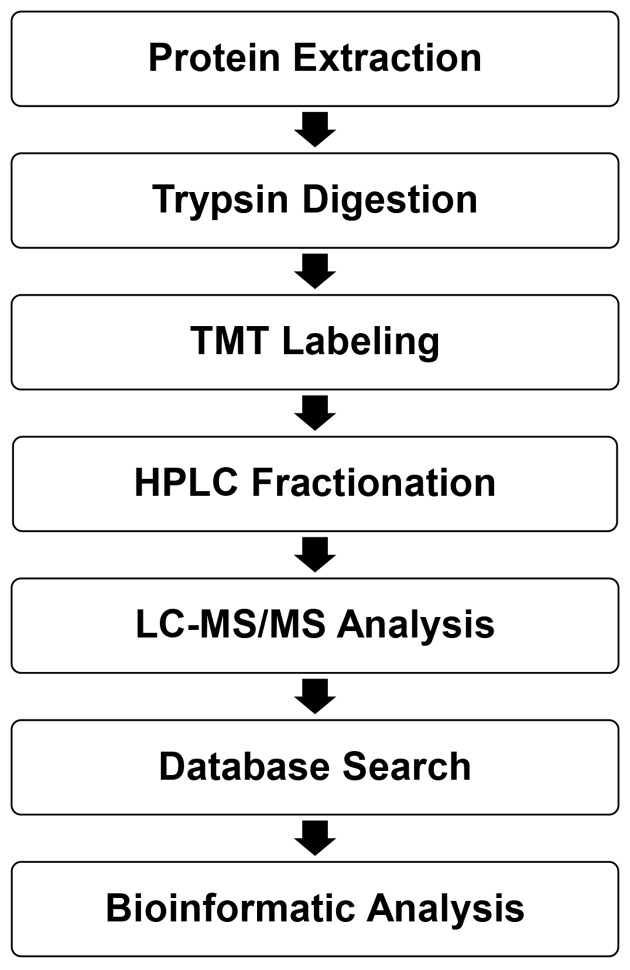
Technical route of quantitative serum proteomics in NPC patients

The experimental procedures are shown in Figure 1.

### Protein extraction

Serum samples were collected. Protein concentration was determined using a BCA kit according to the manufacturer’s instructions. First, cellular debris was removed by centrifugation at 12,000 ***g*** at 4°C for 10 min and the supernatants transferred to new tubes. High-abundance proteins were removed with the aid of a Pierce Top 12 Abundant Protein Depletion Spin Column Kit (Thermo Fisher Scientific).

### Trypsin digestion

Prior to digestion, protein solutions were reduced with 5 mM dithiothreitol for 30 min at 56°C, alkylated with 11 mM iodoacetamide for 15 min at room temperature in the dark, and subjected to ultrafiltration three times. Trypsin (Promega, U.S.A.) was added to a final concentration of 15 ng/μl followed by overnight digestion (37°C), and each peptide solution was centrifuged at 12,000 ***g***at 4°C for 15 min and the peptide concentration then determined using the BCA kit according to the manufacturer’s instructions.

### Tandem mass tag labeling

After trypsin digestion, the peptides were desalted on a Strata X C18 SPE column (Phenomenex), vacuum-dried, reconstituted in 0.5 M triethylammonium bicarbonate buffer (TEAB), and processed according to the manufacturer’s protocol. Briefly, one unit of TMT reagent was thawed and reconstituted in acetonitrile. The peptide mixtures were incubated with the reagent for 2 h at room temperature, then pooled, desalted, and dried via vacuum centrifugation.

### High performance liquid chromatography fractionation

Tryptic peptides were fractionated by high-pH reverse-phase high-performance liquid chromatography using an Agilent 300 Extend C18 column (5 μm particle diameter, 4.6 mm internal diameter, 250 mm in length). Briefly, peptides were first separated into 60 fractions using a gradient of 8–32% acetonitrile (pH 9.0) over 60 min, then combined into nine fractions, and dried by vacuum centrifugation.

### LC-MS/MS

Tryptic peptides were dissolved in 0.1% (v/v) formic acid (solvent A) and directly loaded onto an in-house reverse-phase analytical column (15 cm in length and 75 μm in internal diameter). The gradient featured a rise from 6 to 18% solvent B (0.1% [v/v] formic acid in 98% [v/v] acetonitrile) over 40 min and a further rise from 18 to 28% over 12 min, followed by a rise to 80% (v/v) acetonitrile over 4 min and a hold at 80% (v/v) acetonitrile for the last 4 min, at a constant flow rate of 300 nl/min, using an EASY-nLC 1000 UPLC system.

### Database search

MS/MS data were processed using the Maxquant search engine (version 1.5.2.8). Tandem mass spectra were searched against a human database concentrated with a reverse decoy database (https://web.expasy.org/docs/swiss-prot_guideline.html). Trypsin/P was the specified cleavage enzyme; up to two missed cleavages were allowed. The precursor ion mass tolerances were set to 20 and 5 ppm in the first and main searches, respectively, and the mass tolerance to 0.02 Da. Carbamidomethylation of Cys was specified as the fixed modification, and Met oxidation as a variable modification. The false detection rate was adjusted to <1% and the minimum peptide score to >40.

### Statistical analysis

Patients were divided into those who were radioresistant and radiosensitive by the overall reduction (≤50% or >50%, respectively) in tumor extent [18]; we compared their clinical data and serum protein expression levels. Data that were normally distributed are expressed as means ± S.D. and categorical data as counts with percentages. All identified proteins were divided into two groups by the median expression level. Univariate logistic regression was used to explore the relationships amongst clinical parameters, protein expression levels, and overall tumor reductions. All proteins were evaluated in terms of their differential diagnostic utilities. We drew receiver operating characteristic curves and calculated the areas under the curves (AUCs), sensitivities, specificities, Youden indices, and accuracies. Proteins expressed at significant levels were subjected to logistic regression. We created a diagnostic protein panel and evaluated the diagnostic utility thereof as follows: 0.5 < AUC < 0.7, low utility; 0.7 ≤ AUC < 0.9, moderate utility; and AUC ≥0.9, high Utility [19]. A *P*-value <0.05 was considered to reflect significance.

## Results

### General patient characteristics

We enrolled 44 NPC patients, 29 males (65.9%), and 15 females (34.1%). Their general characteristics are listed in Table 1. The mean patient age was 45.6 ± 11.2 years. Of all patients, 47.7% had stage III and 52.3% stage IV disease. Eight patients exhibited differentiated and 36 exhibited undifferentiated pathologies. The radiosensitive group contained 27 (61.4%) patients and the radioresistant group 17 patients (38.6%). Univariate logistic regression identified no significant between-group differences between any clinical parameter or the overall tumor reduction rate (Table 1 and Supplementary Material 1).

**Table 1:** Clinicopathological parameters of included patient with NPC

Parameters	Number (%)
Age	
<40	20 (45.5%)
≥40	24 (54.5%)
Gender	
Male	29 (65.9%)
Female	15 (34.1%)
TNM stage	
III	21 (47.7%)
IV	23 (52.3%)
Pathological type	
Differentiated	8 (18.2%)
Undifferentiated	36 (81.8%)
Prior treatment	
Nasopharynx GTVnx	41.9 ± 25.6
Lymph GTVnx	23.9 ± 38.4
Lymph reduction rate	0.57 ± 0.29
>0.50	26 (59.1%)
≤0.5	18 (40.9%)
Overall reduction rate (%)	0.57 ± 0.23
>0.50	27 (61.4%)
≤0.5	17 (38.6%)

### Differential protein screening

The MS/MS spectral search identified 911 proteins, of which 809 were quantitatable (Supplementary Material 2). Proteins were divided into those expressed at high and low levels by reference to the median expression level. Univariate logistic regression identified 20 candidate predictive proteins (Table 2). The serum expression levels of ‘secreted protein acidic and cysteine-rich’ (SPARC), ‘serpin family D member 1’ (SERPIND1), ‘complement C4B’ (C4B), ‘pro-platelet basic protein’ (PPBP), ‘podocalyxin-like protein’ (PODXL), ‘serglycin’ (SRGN), ‘peptidylprolyl isomerase B’ (PPIB), ‘S100 calcium-binding Protein A4’ (S100A4), and ‘cathepsin F’ (CTSF) were significantly higher in the radioresistant than in the radiosensitive group (all *P*<0.05). The ‘endoplasmic reticulum aminopeptidase 1’ (ERAP1), ‘vitamin D-binding protein’ (GC), ‘inter-α-trypsin inhibitor heavy chain 1’ (ITIH1), ‘neuropilin 1’ (NRP1 or C1R), ‘multiple inositol-polyphosphate phosphatase 1’ (MINPP1), ‘coagulation factor XIII A chain’ (F13A1), ‘complement C1qB chain’ (C1QB), ‘inter-α-trypsin inhibitor heavy chain 2’ (ITIH2), ‘insulin-like growth factor binding protein 6’ (IGFBP6), and ‘family with sequence similarity 173 member A’ (FAM173A) proteins were significantly down-regulated in the radioresistant compared with the radiosensitive group (all *P*<0.05). We subjected the levels of the 20 candidate proteins to stepwise multivariate logistic regression. Finally, five proteins were used to create the optimal model (Table 3): SPARC (*P=*0.032), SERPIND1 (*P=*0.040), C4B (*P=*0.017), PPIB (*P=*0.042), and FAM173A (*P=*0.017).

**Table 2:** Univariate logistic regression of groups of proteins for overall reduction rate

Protein	Expression level	β	SE	Waldχ^2^	*P*	OR	95% CI	
SPARC	Up-regulation	1.872	0.703	7.098	0.008	6.50	1.64	25.76
ERAP1	Down-regulation	−1.741	0.679	6.572	0.010	0.18	0.05	0.66
SERPIND1	Up-regulation	1.709	0.697	6.015	0.014	5.53	1.41	21.66
GC	Down-regulation	−1.569	0.671	5.468	0.019	0.21	0.06	0.78
C4B	Up-regulation	1.569	0.671	5.468	0.019	4.80	1.29	17.88
ITIH1	Down-regulation	−1.569	0.671	5.468	0.019	0.21	0.06	0.78
PPBP	Up-regulation	1.553	0.693	5.023	0.025	4.73	1.22	18.39
PODXL	Up-regulation	1.406	0.665	4.472	0.035	4.08	1.11	15.02
NRP1	Down-regulation	−1.406	0.665	4.472	0.035	0.25	0.07	0.90
C1R	Down-regulation	−1.406	0.665	4.472	0.035	0.25	0.07	0.90
SRGN	Up-regulation	1.406	0.665	4.472	0.035	4.08	1.11	15.02
PPIB	Up-regulation	1.406	0.665	4.472	0.035	4.08	1.11	15.02
CTSF	Up-regulation	1.406	0.665	4.472	0.035	4.08	1.11	15.02
MINPP1	Down-regulation	−1.406	0.665	4.472	0.035	0.25	0.07	0.90
F13A1	Down-regulation	−1.299	0.651	3.979	0.046	0.27	0.08	0.98
C1QB	Down-regulation	−1.299	0.651	3.979	0.046	0.27	0.08	0.98
ITIH2	Down-regulation	−1.299	0.651	3.979	0.046	0.27	0.08	0.98
IGFBP6	Down-regulation	−1.299	0.651	3.979	0.046	0.27	0.08	0.98
S100A4	Up-regulation	1.299	0.651	3.979	0.046	3.67	1.02	13.14
FAM173A	Down-regulation	−1.299	0.651	3.979	0.046	0.27	0.08	0.98

CTSF, cathepsin F; S100A4, S100 calcium binding protein A4.

**Table 3:** Multivariate logistic regression by stepwise for overall reduction rate

Gene	β	SE	Wald χ^2^	*P*	OR	95% CI	
SPARC	3.025	1.406	4.627	0.032	20.6	1.308	324.4
SERPIND1	2.582	1.258	4.215	0.040	13.22	1.124	155.4
C4B	3.723	1.561	5.692	0.017	41.39	1.943	881.7
PPIB	3.103	1.523	4.150	0.042	22.27	1.125	440.8
FAM173A	−4.248	1.778	5.707	0.017	0.014	<0.001	0.466
Intercept	−4.798	1.574	9.291	0.002			

PPIB, peptidylprolyl isomerase B.

### The NPC diagnostic utilities of serum proteins

**Figure 2:**
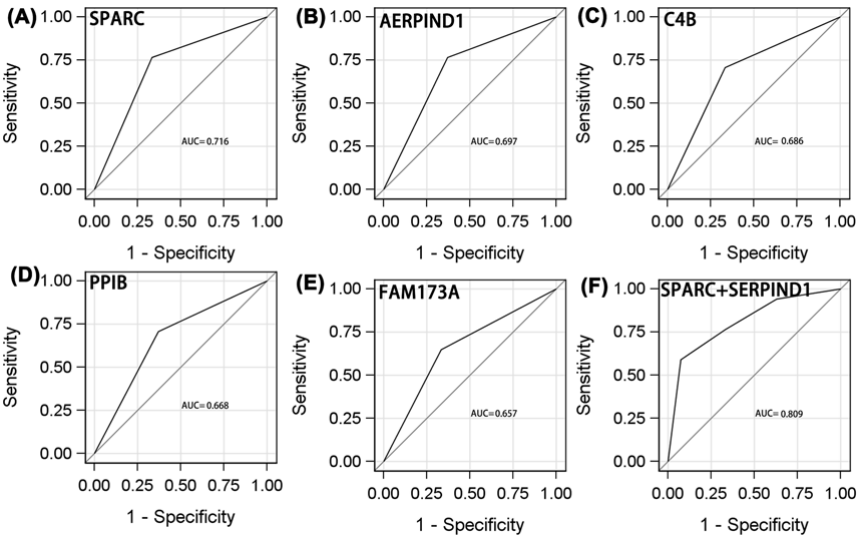
ROC analyses of protein to distinguish adverse efficacy from NPC patients who receive radiotherapy ((**A**) SPARC; (**B**) AERPIND1; (**C**) C4B; (**D**) PPIB; (**E**) FAM173A; (**F**) SPARC+ SERPIND1).

We assessed the differential diagnostic utilities of each protein and combinations of two, three, four, and five proteins with the largest AUCs. For all patients, the AUCs for SPARC, SERPIND, C4B, PPIB, and FAM173A were 0.716 (95% CI: 0.574–0.881), 0.697 (95% CI: 0.837–0.858), 0.686 (95% CI: 0.522–0.850), 0.668 (95% CI: 0.502–0.834), and 0.657 (95% CI: 0.512–0.825), respectively (Figure 2A–E and Table 4). The respective sensitivities and specificities were 0.765 and 0.667, 0.765 and 0.630, 0.706 and 0.667, 0.706 and 0.630, and 0.647 and 0.667. Of the individual proteins, SPARC exhibited the highest diagnostic utility.

**Table 4:** Diagnostic sensitivity and specificity for groups of serum proteins in NPC efficacy

Variables in Model	Sensitivity	Specificity	Youden index	AUC	Correct rate
SPARC	0.765	0.667	0.431	0.716	0.705
SERPIND1	0.765	0.630	0.394	0.697	0.681
C4B	0.706	0.667	0.372	0.686	0.681
PPIB	0.706	0.630	0.336	0.668	0.659
FAM173A	0.647	0.667	0.313	0.657	0.659
SPARC SERPIND1	0.588	0.926	0.514	0.809	0.795
SPARC FAM173A	0.765	0.667	0.431	0.804	0.727
SERPIND1 C4B	0.529	0.926	0.455	0.792	0.772
SPARC C4B	0.765	0.667	0.431	0.794	0.750
SERPIND1 PPIB	0.765	0.630	0.394	0.785	0.727
C4B FAM173A	0.412	0.963	0.374	0.777	0.750
C4B PPIB	0.787	0.588	0.588	0.769	0.841
SPARC PPIB	0.418	0.765	0.431	0.770	0.705
PPIB FAM173A	1.00	0.407	0.407	0.754	0.681
SERPIND1 FAM173A	0.765	0.630	0.394	0.761	0.704
SPARC C4B FAM173A	0.882	0.778	0.660	0.878	0.818
SERPIND1 C4B PPIB	0.765	0.815	0.579	0.862	0.795
C4B PPIB FAM173A	0.706	0.963	0.669	0.842	0.864
SPARC SERPIND1 C4B	0.941	0.741	0.682	0.883	0.818
SPARC SERPIND1 FAM173A	0.882	0.778	0.660	0.877	0.818
SERPIND1 C4B FAM173A	0.824	0.741	0.564	0.852	0.795
SPARC SERPIND1 PPIB	0.882	0.778	0.660	0.867	0.818
SPARC PPIB FAM173A	0.941	0.704	0.645	0.795	0.862
SERPIND1 PPIB FAM173A	0.941	0.704	0.645	0.795	0.855
SPARC C4B PPIB	0.647	0.889	0.535	0.815	0.795
SERPIND1 C4B PPIB FAM173A	0.706	0.963	0.669	0.914	0.863
SPARC SERPIND1 C4B FAM173A	0.824	0.963	0.786	0.928	0.909
SPARC C4B PPIB FAM173A	0.882	0.889	0.771	0.939	0.886
SPARC SERPIND1 C4B PPIB	0.941	0.852	0.793	0.886	0.902
SPARC SERPIND1 PPIB FAM173A	0.824	0.926	0.749	0.886	0.922
SPARC SERPIND1 C4B PPIB FAM173A	0.941	0.926	0.867	0.968	0.932

PPIB, peptidylprolyl isomerase B.

**Figure 3:**
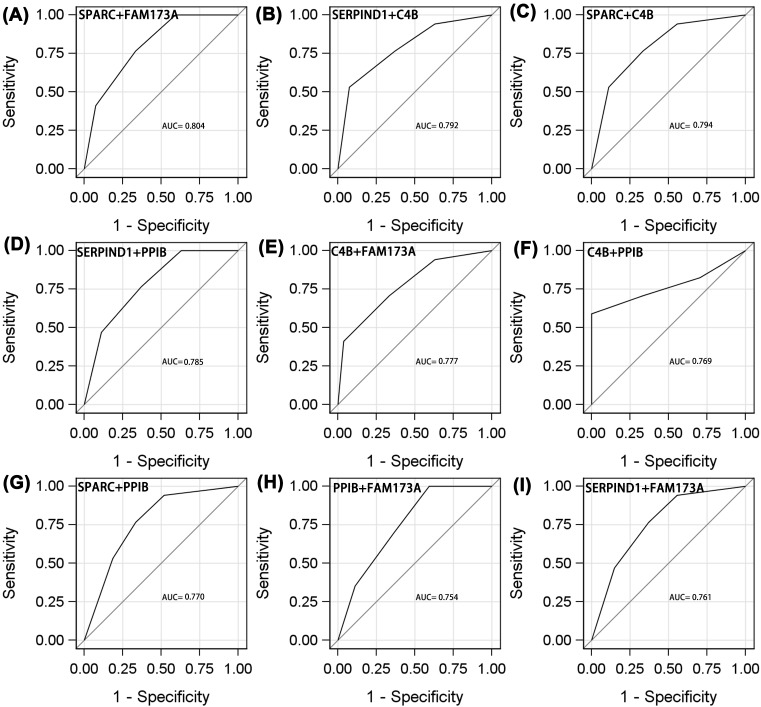
ROC analyses of protein to distinguish adverse efficacy from NPC patients who receive radiotherapy ((**A**) SPARC+FAM173A; (**B**) SERPIND1+C4B; (**C**) SPARC+C4B; (**D**) SERPIND1+PPIB; (**E**) C4B+FAM173A; (**F**) C4B+PPIB; (**G**) SPARC+PPIB; (**H**) PPIB+FAM173A; (**I**) SERPIND1+FAM173A).

Of the combinations of two proteins (Figures 2F and 3A–I), SPARC/SERIND1 exhibited the highest AUC (0.809, 95% CI: 0.674–0.945), sensitivity (0.588), and specificity (0.926). PPIB/FAM173A was of lower diagnostic utility (AUC = 0.754, 95% CI: 0.613–0.894, sensitivity = 1.00, specificity = 0.407).

**Figure 4:**
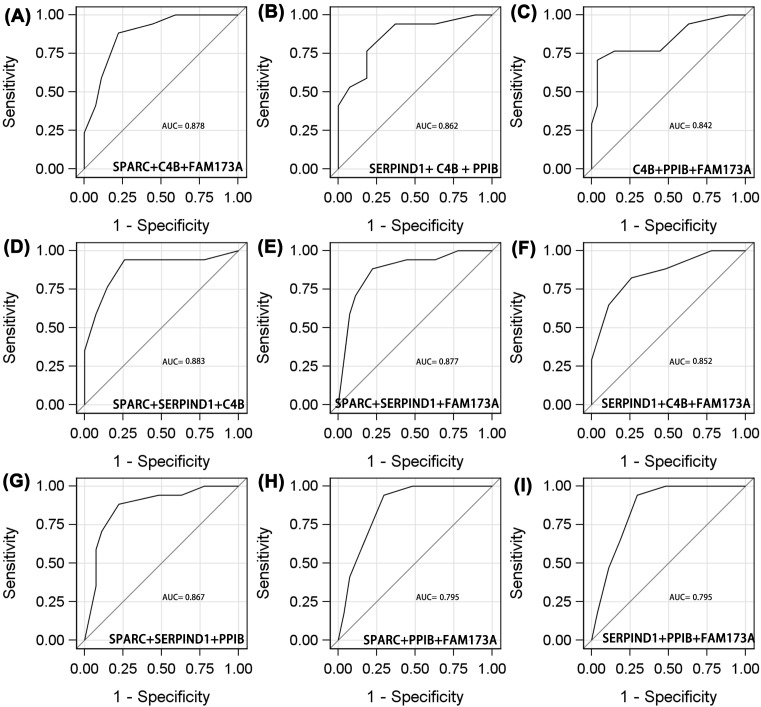
ROC analyses of protein to distinguish adverse efficacy from NPC patients who receive radiotherapy ((**A**) SPARC+C4B+FAM173A; (**B**) SERPIND1+ C4B + PPIB; (**C**) C4B+PPIB+FAM173A; (**D**) SPARC+SERPIND1+C4B; (**E)** SPARC+SERPIND1+FAM173A; (**F**) SERPIND1+C4B+FAM173A; (**G**) SPARC+SERPIND1+PPIB; (**H**) SPARC+PPIB+FAM173A; (**I**) SERPIND1+PPIB+FAM173A).

**Figure 5:**
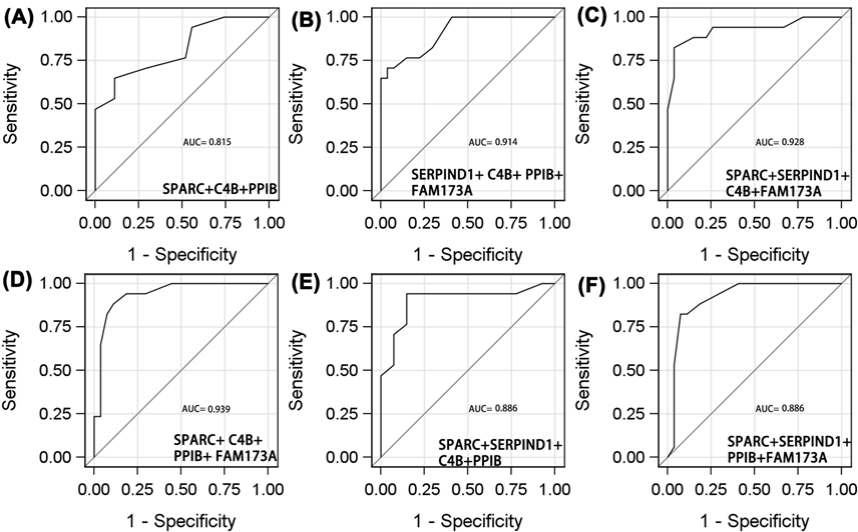
ROC analyses of protein to distinguish adverse efficacy from NPC patients who receive radiotherapy (**A**) SPARC+C4B+PPIB; (**B**) SERPIND1+ C4B+ PPIB+ FAM173A; (**C**) SPARC+SERPIND1+C4B+FAM173A; (**D**) SPARC+ C4B+ PPIB+ FAM173A; (**E**) SPARC+SERPIND1+C4B+PPIB; (**F**) SPARC+SERPIND1+PPIB+FAM173A.

**Figure 6:**
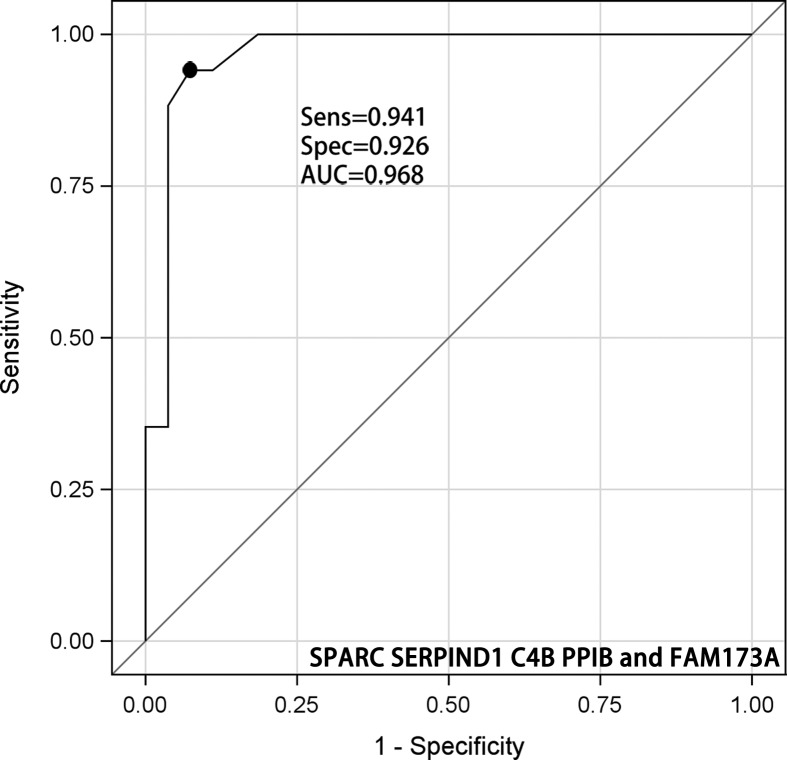
ROC curve for selected model including SPARC SERPIND1 C4B PPIB and FAM173A

Of the three-protein combinations (Figure 4A–I), the diagnostic utility of SPARC/C4B/FAM173A was the same as those of SPARC/SERPIND1/FAM173A and SPARC/SERPIND1/PPIB. The AUCs, sensitivities, and specificities were 0.878, 0.882, and 0.778; 0.877, 0.882, and 0.778; and 0.867, 0.882, and 0.778, respectively. Of the four-protein combinations (Figure 5A–F), SPARC/C4B/PPIB/FAM173A was optimal, with an AUC of 0.939 (95% CI: 0.876–1.000), sensitivity 0.882, and specificity 0.889. The five-protein AUC was 0.968 (95% CI: 0.918–1.000), sensitivity 0.941, and specificity 0.926 (Figure 6). The diagnostic utilities of other protein combinations are shown in Table 4 and Figures 3–5.

## Discussion

In the present study, we identified 874 proteins, 20 of which were differentially expressed in radioresistant and radiosensitive NPC patients. We used a SPARC/SERPIND1/C4B/ PPIB/FAM173A panel to predict the response of NPC to radiotherapy. The panel afforded a sensitivity of 0.941 and a specificity of 0.926 with an AUC of 0.968 and thus effective. To our knowledge, this is the first study to explore the differential diagnostic utilities of serum proteins in terms of NPC radiosensitivity.

NPC is more sensitive to ionizing radiation than other head-and-neck cancers. Radiotherapy is the primary treatment option. Recently, improvements in radiotherapy have enhanced the survival of even patients with advanced disease [20]. However, some NPC patients do not respond to radiotherapy. Local recurrence and distant metastasis attributable to radioresistance impede treatment. Serum biomarkers of NPC radioresistance would be useful in predicting outcomes, facilitating exploration of the underlying molecular mechanisms, and potentially identifying novel therapeutic targets. Many studies have explored the pathogenesis of NPC radioresistance. However, no study has explored changes in serum protein levels. The present results had some differences from previous reports in other cancers [21,22]. Several reasons may explain the differences: (1) different diseases: Li performed analysis in esophageal cancer, Skiöld performed in breast cancer; (2) samples size difference: Li had ten samples, Skiöld had eight radiation-sensitive and nine normo-sensitive, and we have 44 samples; and (3) different definition: Skiöld compared cancer patients after chemoradiotherapy treatment with the healthy control group. Both Li and our study focussed on radiotherapy sensitivity in cancer patients. Li defined radiotherapy sensitivity according to the radiotherapy oncology group. Our study refers to the overall remission rate of tumor volume.

Inflammation is a critical part of a broad biological response that is initiated after cell injury due to infection or sterile damage, such as cell death. These processes are activated by the immune system to terminate or neutralize destructive stimuli and initiate regeneration. Growing evidence has suggested that IR modulates the immune system through up-regulation of inflammatory mediators [23]. The present study also found some differences proteins related to immune system and inflammation such as C1QB, C4B, ITIH1, ITIH2, SERPIND1. These proteins were also reported by Jeonek et al. who explored the changes of the proteome and metabolome levels in the blood of cancer patients treated with radiotherapy [21]. The overall inflammatory factors were increased for cancer patients who received radiotherapy. The equilibrium between pro and anti-inflammatory cytokines just prior to radiotherapy is important, as this status may affect the rumor resistance to radiotherapy [24]. Moreover, radiotherapy also induces pro-inflammatory mechanism in tumor and normal cells after receiving sub-lethal does, primarily mediated through some transcription factors, which was related to carcinogenesis, inflammation and radio resistance [25]. Usually, most inflammatory factors are up-regulated after radiotherapy, such as SERPIND1 and C4B in the present study. However, some inflammatory and immune-related protein were down-regulated such as ITIH1, ITIH2, C1QB. These differential proteins indicated that blood biomarkers are extensive in monitoring the response to radiotherapy.

We found that the expression levels of SPARC, SERPIND1, C4B, PPIB, and FAM173A were significantly higher in radioresistant than in radiosensitive patients. SPARC encodes a cysteine-rich acidic matrix-associated protein located principally in the extracellular space and nucleus. The protein is involved in extracellular matrix synthesis and changes in cell shape. Some studies indicated that SPARC was a tumor suppressor, but others found that the protein gene was associated with tumor metastasis and invasion by changing the cell shape. Three transcriptional variants of the gene encode different protein isoforms. SPARC expression was positively correlated with NPC radioresistance and may affect NPC cell proliferation and growth. SERPIND1 expression was also associated with radioresistance. SERPIND1 belongs to the serpin gene superfamily. The protein is located both extracellularly and in the endoplasmic reticulum. SERPIND1 was reported to be a thrombin inhibitor that interacts with heparin [26]. To date, the role played by SERPIND1 in the context of cancer development remains largely unknown, particularly in NPC. Zhu et al. reported that SERPIND1 was a potential oncogene, triggering ovarian cancer, but the molecular mechanism involved was unclear [27]. High-level SERPIND1 expression was associated with poor prognosis and recurrence in patients with non-small cell lung cancer (NSCLC). The gene promoted the activity of, invasion by, and filopodial dynamics within NSCLC cells by activating the PI3K signaling pathway [28]. High-level serum SERPIND1 expression was observed in patients with B-cell acute lymphoblastic leukemia [29]. In our present study, SERPIND1 was up-regulated in the serum of radioresistant NPC patients. This finding is novel, suggesting a mechanism of radioresistance in some NPC patients, and perhaps serving as a very relevant marker of radioresistance. Further research is required.

The C4B gene lies in the major histocompatibility complex class III region of chromosome 6. The encoded protein locates to the plasma membrane and the extracellular matrix, and (to a minor extent) the nucleus. C4B is a component of the classical activation pathway, and is expressed in a precursor form that is then proteolytically cleaved, prior to secretion, into a trimer composed of α, β, and γ chains. Hepatitis B virus X protein up-regulated C4b-binding protein α synthesis via activation of transcription factor Sp1 to protect hepatoma cells from attacks by complement [30]. The level of the C4B α-chain was significantly higher in patients with pancreatic ductal adenocarcinoma than in healthy controls. The C4B level was of moderate diagnostic utility in terms of pancreatic ductal adenocarcinoma diagnosis (AUC = 0.860), serving as a novel serum biomarker for detecting early stage disease [31]. In the present study, C4B was of moderate utility in terms of diagnosing NPC radioresistance (AUC = 0.686).

PPIB, also termed cyclophilin B, is located principally in the endoplasmic reticulum, and is a cyclosporine-binding protein regulating cyclosporine A-mediated immunosuppression. Recently, it was shown that cyclophilin B induced chemoresistance, interacting with MDM2 to degrade wild-type p53 in colorectal cancer patients [32]. Paul reported that PPIB expression was associated with radioresistance in head-and-neck squamous cell carcinoma, affecting outcomes after radiotherapy. Inhibition of PPIB expression by siRNA increased the radiosensitivity of cancer cells and inhibited DNA repair [33]. We confirmed that these findings using NPC serum samples; the gene may be a useful radiotherapeutic target. FAM173A is located principally in the cytosol but has been little studied. Serum levels of FAM173A were down-regulated in NPC patients; thus, it was negatively associated with radioresistance in NPC patients. FAM173A is found throughout the cytoplasm, and radiotherapy may affect protein synthesis. Further study is needed.

We created a panel of five serum proteins predicting the short-term efficacy of radiotherapy (radiosensitivity) in NPC patients. Compared with other materials, such as miRNAs and lncRNAs, serum proteins are easy to obtain and handle, and our panel, can be simply applied. However, several limitations of the work should be addressed. Liu et al. also identified some serum proteins related with radiosensitive [34]. The present identified proteins were different previous study. The main reasons may be that there are some differences between our study and this article. In our study, patients were divided into those who were radioresistant and radiosensitive by the overall remission (≤50% or >50%, respectively) in tumor extent. We tend to focus on recent effect. The published paper focussed on recurrence and complete remission. The definition of radioresistance may vary if long-term efficacy is considered; more work is needed. Our sample size was small due to the expense of serum proteomics. A larger sample size is required to confirm our findings. Additionally, unexplored factors potentially affecting the results may have been in play.

In summary, we used proteomics to create a panel of five serum proteins (SPARC, SERPIND1, C4B, PPIB, and FAM173A) predicting the radiotherapeutic response in NPC patients. The panel was highly discriminatory. Future studies should confirm our findings in larger patient numbers with longer follow-up, and further explore the mechanism of induced radioresistance.

## Supporting information

**Supplementary Material 1:** 

**Supplementary Material 2:** Univariate logistic regression of clinical parameters for overall reduction rate

## Abbreviations

AUC: area under the curve
C4B: complement C4B
C1QB: complement C1q B chain
ERAP1: endoplasmic reticulum aminopeptidase 1
F13A1: coagulation factor XIII A chain
FAM173A: family with sequence similarity 173 member A
GC: vitamin D binding protein
GTV: gross tumor volume
IGFBP6: insulin like growth factor binding protein 6
ITIH1: inter-α-trypsin inhibitor heavy chain 1
ITIH2: inter-α-trypsin inhibitor heavy chain 2
MINPP1: multiple inositol-polyphosphate phosphatase 1
NPC: nasopharyngeal carcinoma
NRP1: neuropilin 1
NSCLC: non-small cell lung cancer
PODXL: podocalyxin like
PPBP: pro-platelet basic protein
PPIB: peptidylprolyl isomerase B
SERPIND1: serpin family D member 1
SPARC: secreted protein acidic and cysteine rich
SRGN: serglycin
