# Supplementary material for: Serum proteomics identify potential biomarkers for nasopharyngeal carcinoma sensitivity to radiotherapy
Source: Biosci Rep. 2019 May 14;39(5):BSR20190027. doi: 10.1042/BSR20190027 (PMC6522734; doi:10.1042/BSR20190027)
Supplement: Supplementary file 2 [file BSR-2019-0027_suppST1.pdf]

Table 1s Univariate logistic regression of clinical parameters for overall reduction rate

Variable	Beta	SE	Waldχ^2	P	OR	(95%CI)
Gender	-0.508	0.649	0.615	0.433	0.60	0.17-2.14
Age	0.158	0.206	0.588	0.443	1.17	0.78-1.75
Stage	-0.341	0.621	0.301	0.583	0.71	0.21-2.40
differentiation	-0.059	0.806	0.005	0.942	0.94	0.19-4.58
nasopharynx GTVnx	-0.001	0.013	0.010	0.920	1.00	0.97-1.02
Lymph GTVnd	-0.042	0.025	2.811	0.094	0.96	0.91-1.01
Red blood cell	-0.182	0.658	0.077	0.782	0.83	0.23-3.03
White blood cell	0.514	0.656	0.612	0.434	1.67	0.46-6.05
Neutrophil cells	0.879	0.674	1.697	0.193	2.41	0.64-9.03
lymphocyte	-1.348	0.698	3.729	0.054	0.26	0.07-1.02
Hemoglobin	-0.619	0.665	0.867	0.352	0.54	0.15-1.98
Alpha fetoprotein	-0.434	0.661	0.430	0.512	0.65	0.18-2.37
CEA	0.000	0.657	0.000	1.000	1.00	0.28-3.63
CA125	0.000	0.657	0.000	1.000	1.00	0.28-3.63
CA242	-0.251	0.660	0.145	0.703	0.78	0.21-2.84
C19F	0.434	0.661	0.430	0.512	1.54	0.42-5.64
EB virus DNA	-0.956	0.707	1.829	0.176	0.39	0.10-1.54
EAIgA	-0.693	0.949	0.534	0.465	0.500	0.08-3.21
EBVCAIgA	-0.406	0.904	0.201	0.654	0.67	0.11-0.92

* carcinoembryonic antigen, CEA; CA125, Cancer antigen 125; EB, Epstein-Barr virus, EBV
